# Why Canadian MAiD is Not a Medical Procedure

**DOI:** 10.1177/00243639261492161

**Published:** 2026-10-12

**Authors:** Uzair Jamil, Joshua M. Pearce

**Affiliations:** 1 6221 Western University, London, ON, Canada

**Keywords:** Canada, Medical assistance in dying, medical ethics, palliative care, physician-assisted death

## Abstract

Canada introduced Medical assistance in dying (MAiD) in 2016, and it now accounts for more than 5% of all deaths in the country. Although it is framed as a healthcare service, MAiD is fundamentally incompatible with medicine. There are procedural, philosophical, and ethical distinctions between MAiD and palliative care, which are also confirmed by major national and international organizations including the World Health Organization (WHO) and the Canadian Society of Palliative Medicine. MAiD contradicts the foundational principles of medicine, including the Hippocratic Oath's prohibition against administering lethal substances. The intentional killing of a patient, regardless of consent, cannot constitute a therapeutic act. Normalized terminology and systemic underfunding of palliative care obscure these distinctions and risk eroding the physician–patient relationship.

## What is MAID?

Medical assistance in dying (MAiD) was introduced in Canada through Bill C-14 in 2016. It decriminalized assistance in death and describes both voluntary euthanasia as well as assisted suicide (Coelho et al. 2023). Voluntary euthanasia is where a healthcare practitioner directly administers a substance to kill the patient, whereas assisted suicide means providing the patient with a lethal substance to self-administer. In Canada, euthanasia accounts for almost all MAiD deaths (Coelho et al. 2023), and the use of the terminology “MAiD” instead of euthanasia makes it appear to be a form of healthcare service (Elsner et al. 2024). The eligibility criteria of MAiD under Canadian Criminal Code amendments (2016) include being 18 years old, eligible for government-funded health services, and have grievous and incurable/irrecoverable medical condition (Frolic and Oliphant 2022). The person should also make the request for MAiD voluntarily free from external influences as well as provide informed consent after being provided with alternatives that are available to alleviate suffering including palliative care (Frolic and Oliphant 2022). In 2021, through Bill C-7, the eligibility criteria for MAiD were expanded. Two pathways were introduced to receive MAiD: Track 1 referred to MAiD where the patients’ natural death was reasonably foreseeable, and Track 2 for individuals who were suffering from serious illnesses, but their natural death was not reasonably foreseeable (Coelho et al. 2023). The number of MAiD cases have grown rapidly (Figure 1) and account for over 5% of all deaths in Canada (2024).

**Figure 1.**
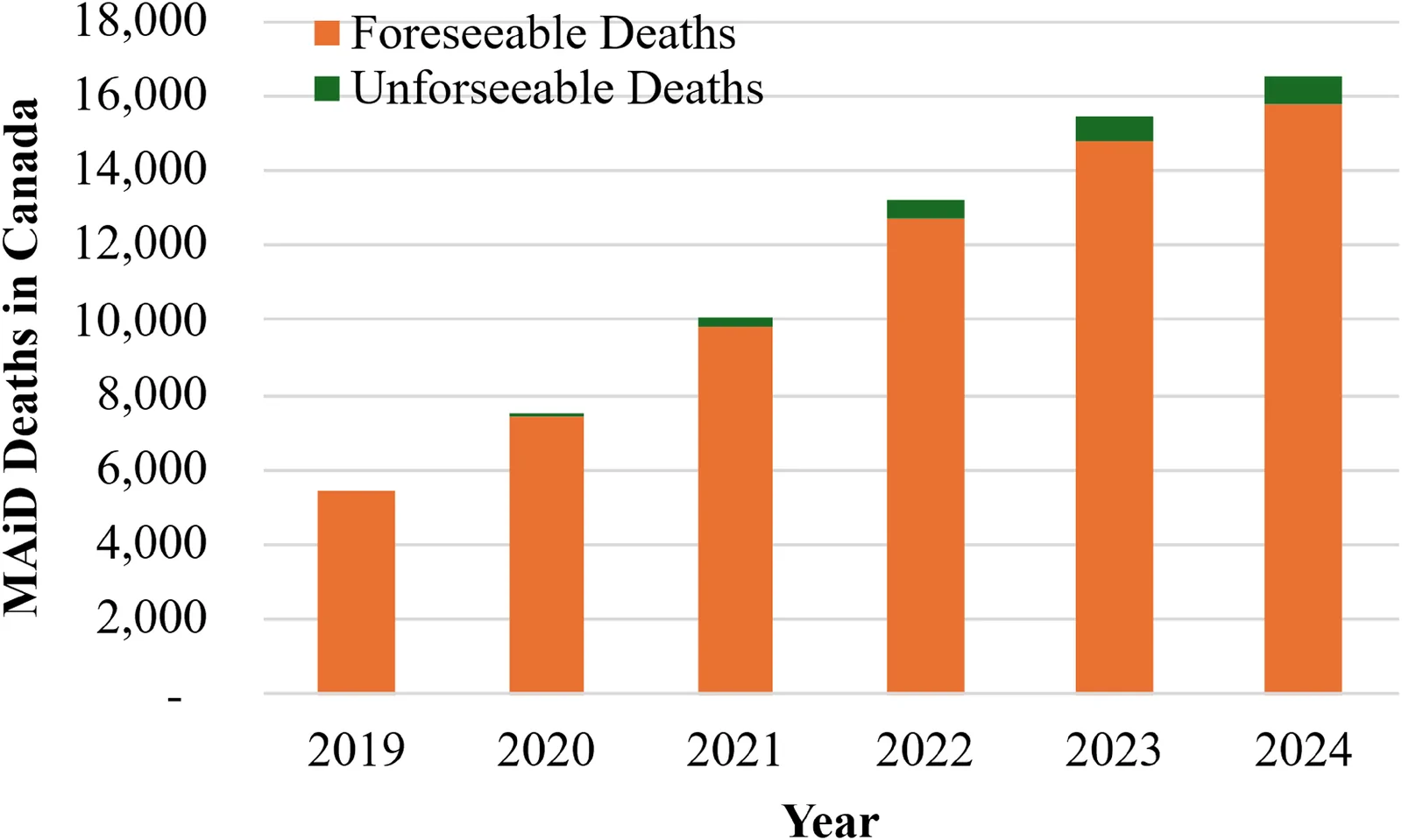
MAiD deaths in Canada over time.

## The Procedure of MAiD

For administering MAiD, two intravenous lines are inserted into the patient (Crumley et al. 2021). The clinician then draws up and administers four syringes:

1. a sedative (midazolam with a median dose of 10 mg used in 91.4% of cases) causing the person to fall asleep in 30 to 60 seconds,
2. a numbing agent (lidocaine with a median dose of 40 mg used in 69.9% of cases) circumventing discomfort from the following injections,
3. a coma-inducing agent (propofol with a median dose of 1,000 mg used in 98% of cases), and
4. a muscle relaxant (rocuronium with a median dose of 200 mg used in 90.8% of cases) stopping breathing and cardiac function within 2 to 6 minutes.

Once the injections are completed, the clinician records the chemicals administered (substance and amount), the time they were injected, the time of patient's death as well as completes the death certificate. Clinicians can bill the cost to Medical Service Insurance and send travel reimbursement claims to health authorities (Crumley et al. 2021).

The chemical cost of MAiD is estimated to be C$1,324 (C$662 for the primary and the backup kit each), which is about half of the total cost. The government economic savings of MAiD and its ethical implications have been examined in previous studies (Jamil and Pearce 2025).

## What is Palliative Care?

In Canada, Health Canada developed a framework of palliative care in 2018 using the World Health Organization's (WHO) definition, which defines palliative care as an approach that improved patients’ and their families’ life quality who experience life threatening ailments. According to the International Association for Hospice and Palliative Care (IAHPC), palliative care is the active overall care of individuals with serious health related suffering (Radbruch et al. 2020).

Palliative care intends to prevent, identify, assess, and manage physical problems including pain and other distressing symptoms including mental, spiritual and social. It also supports individuals to live a fulfilling life until death. It provides support to the patient's family and the caregivers. Moreover, palliative care is not limited only to end-of-life care; it is applicable during the entire course of illness according to one's need and can be performed concomitantly with disease-modifying therapies as required (Radbruch et al. 2020). Also, it is provided taking into consideration the beliefs and traditions of the patient (Radbruch et al. 2020). Research has also shown positive influence of palliative care on alleviating symptom burden and improving quality of life, while being cost-effective at the same time.

The fundamental principle associated with palliative care (that it neither rushes nor slows down death (Radbruch et al. 2020)) contradicts MAiD, which brings about premature death. Under Canadian law, patients must be informed of available palliative care options before consenting to MAiD, which makes it appear that MAiD is a medical procedure and that patients have palliative care as an option or complementary to MAiD.

## Why MAiD is Not Palliative Care

Firstly, the two approaches differ in intentions: MAiD hastens death, while the palliative care neither hastens nor postpones it (Radbruch et al. 2020). Palliative care's objective is to provide relief from suffering while the individual lives. MAiD, in stark contrast, is the intentional killing of the patient (Shariff and Gingerich 2018). The difference is also philosophical where palliative care not only focuses on the physical aspect, but also on the psychological, social, and spiritual dimensions of the medical condition (Radbruch et al. 2020). Palliative care considers dying as a normal component of life and assists individuals in their lives and death. Multiple national and international organizations have concluded that MAiD is not part of palliative care:

- WHO (WHO 2026)
- IAHPC (De Lima et al. 2017)
- European Association for Palliative Care (Radbruch et al. 2016)
- Canadian Hospice Palliative Care Association (CHPCA and CSPCP 2019*a*; 2019*b*)
- Canadian Society of Palliative Medicine (CHPCA and CSPCP 2019*a*; 2019*b*)

In addition, a number of associations and organizations have opposed euthanasia or physician-assisted suicide (PAS) (Barsness et al. 2020), including:

- World Medical Association (World Medical Association 2019)
- American College of Pediatricians (Vizcarrondo 2014)
- American College of Physicians (Snyder Sulmasy and Mueller 2017)
- American Medical Association (American Medical Association 2026*a*, 2026*b*)
- American Society of Anesthesiologists (American Society of Anesthesiologists 2021)
- National Hospice and Palliative Care Organization (National Hospice & Palliative Care Organization 2005)

Interestingly, palliative care is not considered a right in Canada, whereas MAiD is, which has led to inconsistency in access to the two procedures. Only 59.5% of Canadian patients had access to palliative care before requesting MAiD, while only 27.4% had community palliative care physician (Munro et al. 2020). Unlike MAiD, palliative care is not universally recognized as an insured health service under the Canada Health Act (Standing Committee on Health 2020). Universal access to palliative care has remained a challenge in Canada, and there have been calls to incorporate its standards into the Canada Health Act (Morrison 2018). The Canadian parliamentary budget office has indicated potential healthcare savings from MAiD, reflecting a troublesome incentive to not finance palliative care (Coelho et al. 2023). Through the use of softened terminologies and by presenting MAiD as a form of therapy, the moral gravity of the subject is obscured and the procedure of deliberately killing patients is normalized (Elsner et al. 2024). Individuals have raised concerns feeling scared to request medical help as they were anxious that the physicians would introduce MAiD to them (Coelho et al. 2023). A contrast also exists regarding clinical approaches to suffering, where MAiD does not aim to treat any disease, illness, or conditions; rather it eliminates the patient. This is contrary to any therapeutic procedure (Nielsen 2021). Moreover, in the context of MAiD, suffering is defined subjectively in Canada, meaning that it is for the patient to claim if the suffering is intolerable, and no further evidence is needed. There is also no requirement for the clinicians to agree that there are other treatment options available before administering MAiD (Nielsen 2021; Parliament of Canada 2026).

Multiple safeguards concern with regards to MAiD have already been document in Ontario Chief Coroner's MAiD Death Review Committee. These include lax interpretations of legislative criteria, unwarranted influence to die, as well as clinical and systematic impediments. Individuals waiting to receive medical treatment have been subjected to MAiD (Coelho et al. 2023), reflecting the gravity of the problem and a comparison to palliative care where such instance would never occur.

## Why MAiD is Not a Medical Procedure

MAiD, in its essence, is an act of killing, as evident from the lethal chemical dosages provided during the procedure with the intention of causing death. There is no need for any medical training to kill with lethal injections. Framing MAiD as a part of medicine also threatens the relationship between a physician and the patient who considers the physician as a healer (Snyder and Sulmasy 2001). One of the most contentious moral dilemmas of medicinal profession is iatrogenic harm due to role conflation. One who knows how to heal, also knows how to hurt.

MAiD contradicts the foundational principle of the Hippocratic Oath, which explicitly states not to administer a deadly drug to anyone or suggest such a plan (Antoniou et al. 2010). The practice of MAiD clearly goes against the Hippocratic Oath, which stops the physician from performing any act that would cause a patient to die (Antoniou et al. 2010). The Hippocratic Oath clarifies the role of physician as a healer, whereas MAiD obscures it by legally enabling physicians to kill their patients. It is also important to highlight that the medical prohibition to MAiD does not need to arise from religious grounds, but simply the definition of the purpose of medicine and logic. Since MAiD eliminates the person and destroys the patient's body, it is not proper medicine, the goal of which is to protect, promote, and maintain an individual's physical and mental health (Avci 2022). To provide relief from suffering is one of the goals of medicine, however; to kill a patient in order to reduce suffering is a logical absurdity.
